# Qualitative Evaluation of an Artificial Intelligence–Based Clinical Decision Support System to Guide Rhythm Management of Atrial Fibrillation: Survey Study

**DOI:** 10.2196/36443

**Published:** 2022-08-11

**Authors:** John Stacy, Rachel Kim, Christopher Barrett, Balaviknesh Sekar, Steven Simon, Farnoush Banaei-Kashani, Michael A Rosenberg

**Affiliations:** 1 Department of Medicine University of Colorado Aurora, CO United States; 2 Colorado Center for Personalized Medicine University of Colorado Anschutz Medical Campus Aurora, CO United States; 3 Division of Cardiology University of Colorado Anschutz Medical Campus Aurora, CO United States; 4 Department of Computer Science University of Colorado Denver, CO United States

**Keywords:** Clinical decision support system, machine learning, supervised learning, reinforcement learning, atrial fibrillation, rhythm strategy

## Abstract

**Background:**

Despite the numerous studies evaluating various rhythm control strategies for atrial fibrillation (AF), determination of the optimal strategy in a single patient is often based on trial and error, with no one-size-fits-all approach based on international guidelines/recommendations. The decision, therefore, remains personal and lends itself well to help from a clinical decision support system, specifically one guided by artificial intelligence (AI). QRhythm utilizes a 2-stage machine learning (ML) model to identify the optimal rhythm management strategy in a given patient based on a set of clinical factors, in which the model first uses supervised learning to predict the actions of an expert clinician and identifies the best strategy through reinforcement learning to obtain the best clinical outcome—a composite of symptomatic recurrence, hospitalization, and stroke.

**Objective:**

We qualitatively evaluated a novel, AI-based, clinical decision support system (CDSS) for AF rhythm management, called QRhythm, which uses both supervised and reinforcement learning to recommend either a rate control or one of 3 types of rhythm control strategies—external cardioversion, antiarrhythmic medication, or ablation—based on individual patient characteristics.

**Methods:**

Thirty-three clinicians, including cardiology attendings and fellows and internal medicine attendings and residents, performed an assessment of QRhythm, followed by a survey to assess relative comfort with automated CDSS in rhythm management and to examine areas for future development.

**Results:**

The 33 providers were surveyed with training levels ranging from resident to fellow to attending. Of the characteristics of the app surveyed, safety was most important to providers, with an average importance rating of 4.7 out of 5 (SD 0.72). This priority was followed by clinical integrity (a desire for the advice provided to make clinical sense; importance rating 4.5, SD 0.9), backward interpretability (transparency in the population used to create the algorithm; importance rating 4.3, SD 0.65), transparency of the algorithm (reasoning underlying the decisions made; importance rating 4.3, SD 0.88), and provider autonomy (the ability to challenge the decisions made by the model; importance rating 3.85, SD 0.83). Providers who used the app ranked the integrity of recommendations as their highest concern with ongoing clinical use of the model, followed by efficacy of the application and patient data security. Trust in the app varied; 1 (17%) provider responded that they somewhat disagreed with the statement, “I trust the recommendations provided by the QRhythm app,” 2 (33%) providers responded with neutrality to the statement, and 3 (50%) somewhat agreed with the statement.

**Conclusions:**

Safety of ML applications was the highest priority of the providers surveyed, and trust of such models remains varied. Widespread clinical acceptance of ML in health care is dependent on how much providers trust the algorithms. Building this trust involves ensuring transparency and interpretability of the model.

## Introduction

An estimated 2.3 million Americans harbor a diagnosis of atrial fibrillation (AF), and that number is expected to grow to 10 million by 2050 [1,2]. Reduction in mortality and morbidity in patients with AF is predominantly achieved by reducing stroke risk via anticoagulation (AC) [3]. However, AC does not contribute to treating the symptoms of AF or mitigating the long-term effects of living with AF, rather than affecting the sinus rhythm (SR), such as AF-induced cardiomyopathies [4]. Hence, a decision of rate control versus rhythm control must be made. Rate control involves increasing the ventricular filling time by decreasing the ventricular rate, whereas rhythm control involves re-establishment of the SR through some combination of external cardioversion, antiarrhythmic medications, or catheter ablation. The decision of rate versus rhythm control is one that has been studied for years. The data show us that there is no one-size-fits-all answer to this question, and the optimal strategy is highly reliant on individual characteristics and comorbidities of patients with AF [5-16]. The variability of these data and the differences in efficacy in various subsets of the population render the rhythm versus rate control decision a very individualized one and one that lends itself to the aid of clinical decision support systems (CDSSs).

CDSSs encompass a wide array of tools designed to augment and improve clinical outcomes [17]. These tools vary from broad aids such as literature databases [18], to decision trees that help with diagnosis [19-22], to risk stratification tools including the CHA_2_DS_2_Vasc and HEART scores, which estimate stroke risk in patients with AF and 6-week increase in adverse major cardiac events, respectively [23-28]. Although CHA_2_DS_2_Vasc and HAS-BLED (Hypertension, Abnormal renal/liver function, Stroke, Bleeding history or predisposition, Labile International Normalized Ratio, Elderly, Drugs/alcohol concomitantly) [29] scores (the HAS-BLED score estimates risk of major bleeding for patients on AC) have been helpful in determining which patients with AF need AC, there is no CDSS to our knowledge that has been designed to help make the less straightforward AF decision concerning rate versus rhythm control. For more than a decade, the focus in CDSS has been on the development of computerized algorithms to aid in decision-making or development of computerized CDSSs (CCDSSs). A CCDSS harnesses the massive data pool, that is, the electronic health record (EHR), along with advanced computing to aid providers in making complex decisions. CCDSS development is an active field of research focusing on Bayesian networks (BN), machine learning (ML), and artificial neural networks (ANN), but clinical acceptance has lagged behind.

In this study, we introduce a novel ML framework used to create a CCDSS for rhythm management in patients with AF. The QRhythm application is a learning CCDSS that utilizes a 2-stage ML model to identify the optimal rhythm management strategy in a given patient on the basis of a set of clinical factors, in which the model first uses supervised learning (SL) to predict the actions of an expert clinician, and then applies reinforcement learning (RL) to identify the best strategy to obtain the best clinical outcome.

**Figure 1:**
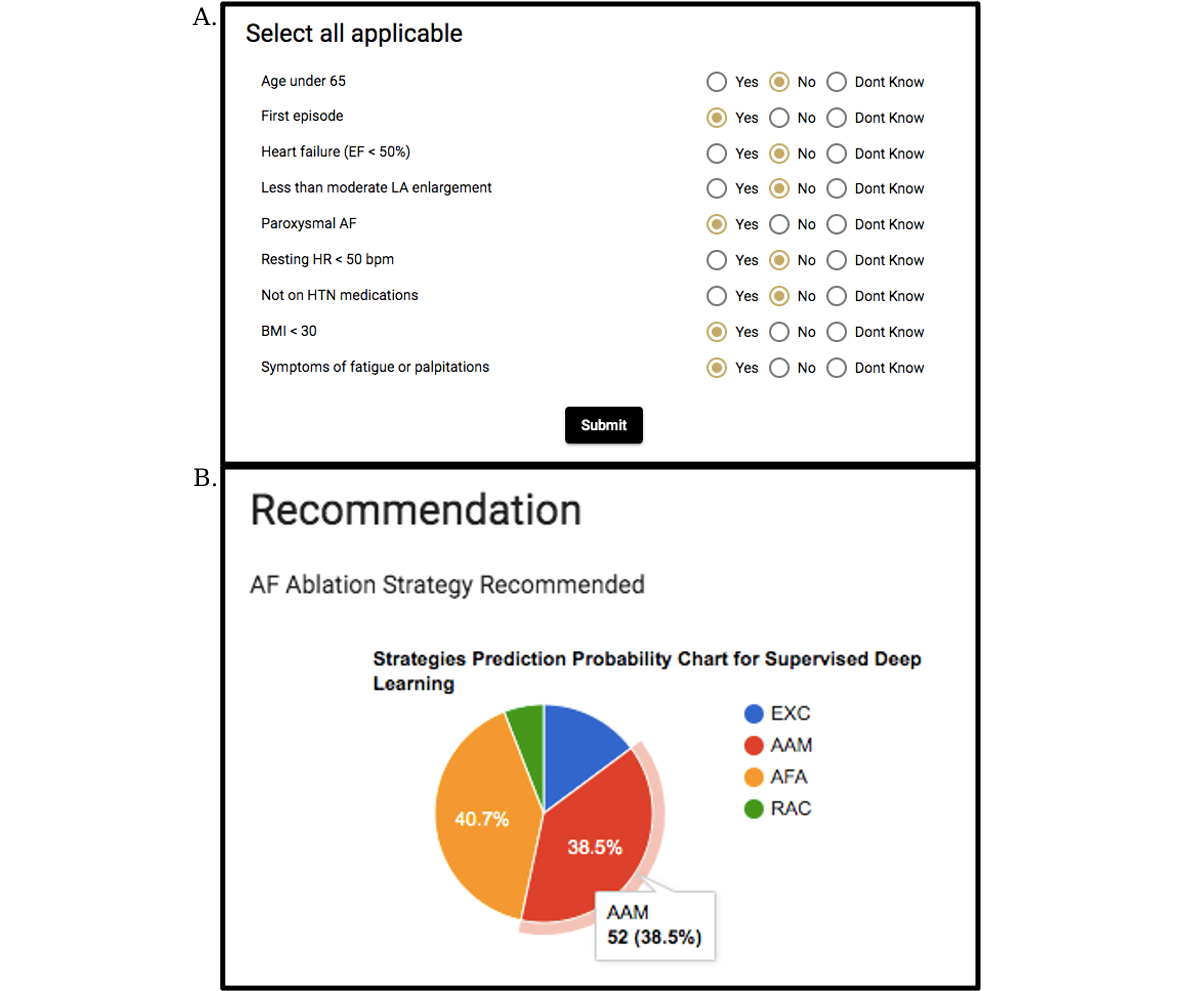
(A) Screenshot of the data input page of QRhythm. (B) Screenshot of the data output page of QRhythm. AAM: antiarrhythmic medication; AF: atrial fibrillation; AFA: atrial fibrillation ablation; EF: ejection fraction; HTN: hypertension; LA: left atrial; HR: heart rate; EXC: external cardioversion; RAC: rate control.

Providers are asked to input data pertaining to the patient in question, including age, duration of AF, history of heart failure, diagnosis of left atrial enlargement, resting heart rate, diagnosis of hypertension, BMI, and symptoms associated with their AF. Based on the answers to these questions, the algorithm predicts the frequency at which an expert would choose different rhythm control strategies including external cardioversion, antiarrhythmic medications, AF ablation, or rate control. Screenshots of the input and output screens of the application are provided in Figures 1A and 1B, respectively.

Both the SL and RL algorithms of the QRhythm application are based on linear regression models, in which the inputs are each weighted toward prediction of each of the 4 possible treatment options: rate control, external cardioversion, antiarrhythmic medication, and ablation. The SL model is trained using stochastic gradient descent, with back-propagation of the gradient of the error, which represents the difference between the predicted treatment and what is actually selected by a treating provider, adjusted by a learning rate of 0.1. The RL model uses an algorithm called Q learning, in which the model is trained in parallel for patients in whom outcome information related to hospitalizations, stroke, and symptomatic recurrence is available in follow-up. Within the Q learning framework, the reward is calculated as –2 × (stroke) – (hospitalization) – (symptomatic recurrence), and is back-propagated across each treatment, after adjustment for a learning rate of 0.01. Owing to the preliminary nature of the RL algorithm, the learning rate is one order of magnitude lower than that used in the SL algorithm. Weight updates are delivered in batches of 8 patients. The QRhythm algorithm was initialized using a combination of big data mining and chart review in the University of Colorado Health system of 100 patients diagnosed with AF, which were used to train the SL algorithm based on the action selected by the treatment provider. The QRhythm application is now being deployed in the clinical setting, where its recommendations are being used to guide rhythm strategy decisions in actual patients. Here, it can continue training of the SL algorithm and begin training the RL algorithm as additional patient information is included.

Over time, the model will use what it has learned in the SL phase as a scaffold to slowly transition from an SL algorithm to an RL one, driven by rewards and punishments based on defined outcomes of hospitalizations, symptoms, quality-of-life scores, and changes in rhythm strategies. Rather than predicting what strategy an expert is most likely to choose, the RL edition will have the ability to suggest actions that an expert may not realize as being beneficial for the patient. Therefore, the RL edition will have the capability to improve outcomes for patients with AF when compared to standard of care. This 2-staged method was utilized by the team at DeepLearning to develop a ML model with the ability to defeat world experts in the game Go [30,31].

We used the QRhythm application as a substrate to analyze the reasons underlying apprehension toward clinical acceptance of CCDSSs in general.

## Methods

### Ethics Approval

This study was approved by the Colorado Multiple Institutional Review Board (#20-2192).

### Qualitative Assessment

Residents, fellows, and attendings were first introduced to the QRhythm app via a brief written tutorial. The providers were asked to examine the app. They were encouraged to use the app in a clinical setting—that is, to help inform a rhythm/rate strategy for a real patient—but this was not required. They then were presented with a survey produced on REDCap. The survey was designed to assess how important the providers thought certain characteristics of the app were. They were asked to rate how important each category was on a scale of 1-5, with 1=unimportant, 2=slightly important, 3=moderately important, 4=important, and 5=very important. Other prompts asked the provider to report how firmly they agreed with a prompt, with 1=“strongly disagree,” 2=“somewhat disagree,” 3=“neutral,” 4=“somewhat agree,” and 5=“strongly agree.” Finally, those who used the app were asked to rank their areas of concern with using the app from 1=highest concern to 5=lowest concern. Data were collected on REDCap and exported to an Excel (Microsoft Inc) spreadsheet for further analysis.

## Results

In total, 33 providers responded to the survey. Seven (21%) of them were attendings (postgraduate year>3), 2 (6%) were fellows, 21 (64%) were residents, and 3 (9%) chose not to identify their provider level. The providers were predominantly either internal medicine residents or attendings (n=27, 82%) with the remainder being either cardiology fellows or attendings (n=6, 18%). We feel that this mix of internists and cardiologists is valuable, as it importantly reflects providers involved in both early-stage (referrals—internists) and later-stage (treatment—cardiologists) management of AF.

**Figure 2:**
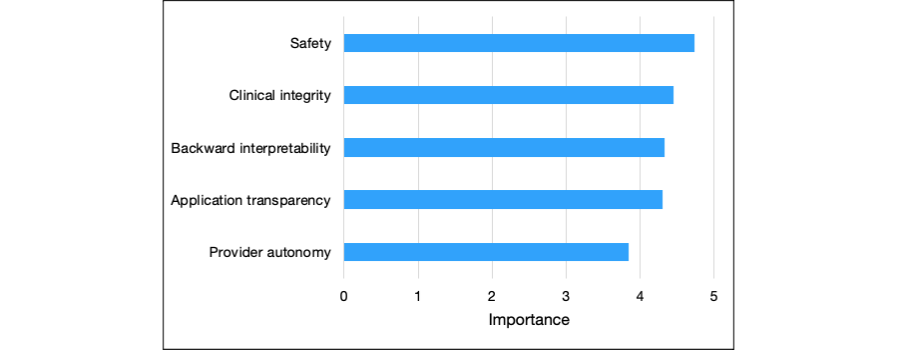
Importance rated by survey respondents on a scale of 1-5, with 1=unimportant, 2=slightly important, 3=moderately important, 4=important, and 5=very important, with regard to safety, clinical integrity, backward interpretability, application transparency, and provider autonomy.

Of the characteristics of the app surveyed, safety was most important to the providers, who reported an average importance rating of 4.7 out of 5 (SD 0.72) in response to the prompt “The model is not recommending anything unsafe or potentially harmful.” This was followed by an importance rating of 4.5 (SD 0.90) for clinical integrity corresponding to the prompt “The information the model is using to make predictions makes sense clinically,” 4.3 (SD 0.65) for backward interpretability (“I know the population in which the model was derived is the same as the one in which I am applying it,”), and 4.3 (SD 0.83) for application transparency (“I understand the reasoning with which the model made its recommendations”). Least important to the providers was provider autonomy with an average importance of 3.85 (SD 0.83) placed on the prompt “I am able to disagree with or challenge the recommendations of the model” (Figure 2).

Of those who responded, 6 providers used the app at least once in the clinical setting; that is, they used the app to aid in developing rhythm control strategies for actual patients. Providers were not required to follow the specific recommendations of QRhythm, but merely to examine agreement and interpretability of the application, as formal testing toward hard clinical outcomes was beyond the scope of this pilot investigation. Apprehension concerning the use of the app clinically varied among these providers. In response to the prompt “I would feel apprehensive about using the QRhythm application,” 1 provider (17%) strongly disagreed, 2 (33%) somewhat disagreed, 2 (33%) were neutral on the subject, and 1 (17%) somewhat agreed. Trust in the app similarly varied. To the prompt “I trust the recommendations provided by the QRhythm app,” 1 provider (17%) somewhat disagreed, 2 (33%) were neutral, and 3 (50%) somewhat agreed. For the most part, providers did not feel intimidated by the app. One provider (17%) strongly disagreed with the statement “the QRhythm application would be intimidating for me to use,” 4 (67%) somewhat disagreed with this statement and 1 (17%) was neutral. To the prompt, “Learning to use the QRhythm application would be easy for me,” 4 providers (67%) strongly agreed, and 2 (17%) somewhat agreed. Finally, in general, providers felt that the QRhythm app would be helpful when taking care of patients with AF. To the prompt “Using the QRhythm application would enhance my effectiveness in patient care and AF management,” 1 provider (17%) strongly agreed, 3 (50%) somewhat agreed, and 2 (33%) were neutral.

These providers were also asked to rank their concerns regarding clinical use of the app from highest to least concern. Four providers (67%) ranked the clinical integrity of recommendations as their highest concern, 1 (17%) ranked ineffectiveness as their highest concern, and 1 (17%) ranked data security as their highest concern.

## Discussion

### Principal Findings

In this study, we examined the provider experience with a novel, AI-based CCDSS for rhythm management of AF. Providers generally found the application easy to use, though trust in the application was variable and apprehension toward its advice remains a concern. Our data show that safety of the recommendations provided by the app was most important to providers. This priority is followed closely by a desire for advice from a CCDSS to make sense clinically, for the provider to have knowledge of the population in which the algorithm was developed, and for transparency of the reasoning with which the app made its decision. Less important in the minds of the providers surveyed was the ability to challenge the decisions made by the app. Of those providers who used the app in the clinical setting, the accuracy of its recommendations ranked highest among their concerns.

### Comparison With Prior Work

Although there are no existing CDSS for rhythm management of AF to our knowledge, there are numerous studies related to specific treatment approaches within the rate versus a rhythm control strategy decision for patients with AF. The AFFIRM trial, published in 2002, showed noninferiority in terms of mortality in rate-controlled patients compared to rhythm-controlled patients, with a trend toward increased mortality in the rhythm control group [5]. This trial subsequently guided rhythm control strategies away from the promise of improvement in mortality, and directed treatment toward improvement in symptoms alone. However, the paradigm for rhythm management may be shifting with the more recent data suggested by the EAST-AFNET 4 trial published in February 2021, which showed that early rhythm control (within a year of AF diagnosis) decreases stroke and cardiovascular mortality compared to a rate control strategy [6].

We surmise that declaring rate control as superior to rhythm control, or vice versa, however, is too sweeping a conclusion to make. Further evaluation of these data reveal that individual characteristics of patients with AF play a large role regarding the success or failure of a chosen strategy. Subsets of patients with AF, such as those with heart failure or left ventricular dysfunction, have been shown to have a mortality benefit from rhythm control [7]. Additionally, the severity of symptoms for patients with AF vary widely, and symptomatic patients experience more relief with rhythm control than with rate control [8-12]. Conversely, factors such as atrial enlargement [13,14], age at onset of AF [15], and duration of AF (paroxysmal vs persistent vs permanent) [16] make rhythm control strategies more difficult to achieve. In other words, the decision about rate or rhythm control in patients is likely to be highly individualized, which raises the possibility that an automated CDSS could provide guidance with AI integration.

Within the category of rhythm control, various rhythm strategies exist including external cardioversion, various antiarrhythmic medications, and AF ablation. The EAST-AFNET 4 trial randomized patients into a rate or rhythm control strategy, but the protocol did not specify which rhythm control strategy was to be pursued. Patients either underwent AF ablation or were started on an antiarrhythmic agent with coincident external cardioversion, but the choice of antiarrhythmic agent was left to the provider [6]. In short, the variability in outcomes based on patient characteristics makes it difficult to generalize and extrapolate data from these studies. It does, however, lend itself to the aid of AI, which can quickly take all of these characteristics into account and provide recommendations accordingly.

The advent of EHR has brought a wealth of data to the fingertips of providers. However, the vastness of these data makes utilizing them time-consuming and clumsy for humans alone. With the aid of AI, CCDSSs aim to create interfaces that are easy to interact with, which condense this plethora of data into easily digestible visualizations to improve the quality of decisions made by providers. Multiple methods have been used to develop such technology, including BNs, ML, and ANNs. While BNs and ANNs are not presently used by QRhythm, these more sophisticated prediction algorithms could easily be incorporated in future versions to include additional types of data, such as electrocardiography (ECG) tracings, patient symptom reports, and clinic notes. The framework we have developed for QRhythm using stochastic gradient descent provides the opportunity for expansion to deep neural networks, for example, which could be used for image recognition or natural language processing to incorporate these additional data types.

ML can be broken down into 3 categories: unsupervised learning (UL), SL, and RL. UL is not used in QRhythm and will therefore not be discussed here.

**Figure 3:**
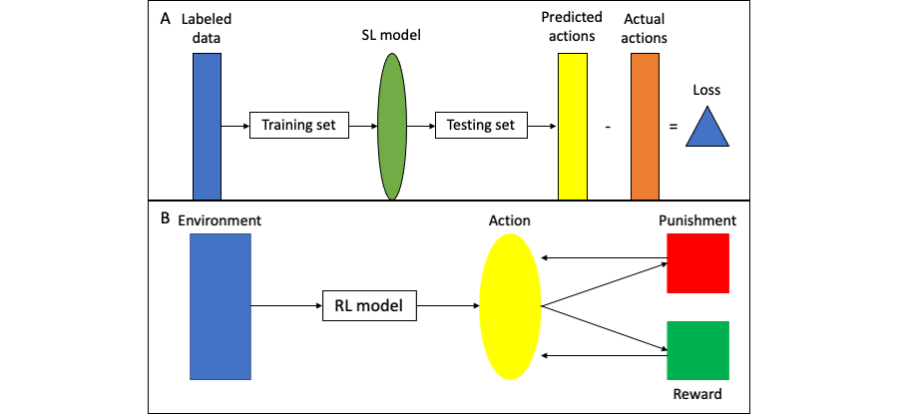
Diagrammatic representations of (A) supervised learning (SL) and (B) reinforcement learning (RL).

In SL, data are input to the model with associated labels; that is, data on patients who underwent a rate control strategy would be labeled as “rate control,” data for those who underwent external cardioversion would be labeled as “external cardioversion,” etc. Through application of various computer algorithms, a model is trained to recognize data associated with these labels in order to predict an outcome using a held-out “training set.” The algorithm is then applied to a second set of data known as the “testing set” or “validation set” to assess its out-of-sample generalizability and efficacy in predicting an outcome. SL comprises the initial strategy for the QRhythm application. Its learning set is a large set of charts of patients with AF. Based on these data, the model has been taught to predict the rhythm strategy most likely to be selected by an expert; for example, an electrophysiologist. SL is designed to mimic the decisions or predictions that would be made by humans. Its efficacy is measured by the difference in the predictions made by the model compared to those made by human experts. This difference is known as the loss function. A loss of zero represents a “perfect” supervised learning model. Therefore, by definition, an SL model can only ever be as effective as the human experts against which it is compared [32-34]. SL models have been used to produce algorithms that accurately interpret ECGs [35,36]. A diagrammatic representation of SL is shown in Figure 3A.

Rather than comparing the model’s performance compared to that of a human expert as in SL, RL is driven by a system of punishments and rewards. RL is an iterative process in which actions are made on the basis of the model’s environment. The results of each action are assessed on the basis of the outcomes of the action. Good outcomes harbor a positive value or “reward”; bad outcomes harbor a negative value or “punishment.” The model takes into account the reward or punishment that results from a certain action and uses this knowledge to inform its next action. RL models are designed to choose actions in order to maximize the reward, thus providing the best outcome possible. As the goal of RL is not merely to mimic humans, but rather to maximize outcomes, it has the potential to outperform human experts. Figure 3B shows a diagrammatic representation of an RL algorithm. An RL model has been used to aid in dosing decisions during dofetilide loading for patients with AF [37].

The advantage of ML in health care is clear; advanced computation allows for analysis of unfathomable amounts of data in infinitesimal amounts of time, which is something the most adept provider simply cannot physically achieve. More data lead to a more informed decision and, therefore, better outcomes [18,38-41]. However, the power and accuracy of ML models are irrelevant if their use is not widely adopted. AI in health care is a robust area of research, but its acceptance in everyday clinical practice lags behind. Two key obstacles standing between the development of AI for health care and its clinical use are model interpretability and trust in the technology.

Interpretability can be broken down into forward or mechanistic interpretability and backward or post hoc interpretability [42]. Forward interpretability represents the ability for a provider to walk through the input portion of the model (ie, its usability). All QRhythm users reported that using the app would be easy for them, indicating good forward interpretability. Backward interpretability represents the ability of a user to easily identify the reasoning behind the decision made by the model. Our survey respondents rated the importance of backward interpretability as 4.3 out of 5 (scale 1-5). Backward interpretability of QRhythm was less convincing; 1 (17%) user reported not understanding the reasoning underlying the decisions made by QRhythm well, 3 (50%) were neutral on the subject, and 2 (33%) reported understanding the reasoning somewhat well.

Maybe the largest obstacle preventing clinical acceptance of AI in health care is trust. ML models require complex mathematics to be functional. As a result, the methodology underlying their decision-making is inherently murky for nonexperts in the field, leading to mistrust by those who do not understand their mechanisms. A study concerning patient apprehension toward AI in health care highlighted that one factor integral to trust in AI was safety of the recommendations made by the model [43]. These concerns were shared by our respondents who rated patient safety as the most important characteristic of the app with an importance rating of 4.7 out of 5. Asan et al [44] highlighted the importance of the transparency of a model when gaining trust. Given the black box nature of these models, achieving transparency is a difficult task. To do so, steps such as furthering education concerning ML in the health care field and providing accessible and understandable explanations of models should be taken.

### Limitations

QRhythm is in the beta testing phase of operation and currently relies heavily on the SL model for recommendations owing to the need for interactive training of RL. Time and increased deployment are needed to improve the accuracy of the recommendations of the model, and additional research is needed to identify a training environment that is safe but also provides meaningful opportunities to apply a computer algorithm in clinical care decisions. Importantly, our work has uncovered the challenges with integration of software development lifecycles, which generally proceed best in a bottom-up, just-in-time development life cycle as employed in the Agile development process, with the necessary top-down guidance from clinical studies and expert opinion. Development of a CDSS that not only provides usability but also meaningful predictions toward an improvement in clinical outcomes is the ultimate goal, and this work represents an important first step.

### Conclusions

In summary, we introduced a novel ML-based model first utilizing SL and then RL to aid in the decision-making process for rhythm strategy for patients with AF. We asked providers to respond to a survey to assess apprehensions regarding the acceptance of such a model for widespread use in clinical practice. Our results show that interpretability and trust of a model are key to acceptance, and providing transparent explanations underlying model reasoning and ensuring the safety of model recommendations are key aspects to improving interpretability and trust.

## Abbreviations

AC: anticoagulation
AF: atrial fibrillation
AI: artificial intelligence
ANN: artificial neural network
BN: Bayesian network
CCDSS: computerized clinical decision support system
CDSS: clinical decision support system
ECG: electrocardiography
HAS-BLED: Hypertension, Abnormal renal/liver function, Stroke, Bleeding history or predisposition, Labile International Normalized Ratio, Elderly, Drugs/alcohol concomitantly
EHR: electronic health record
ML: machine learning
RL: reinforcement learning
SL: supervised learning
SR: sinus rhythm
UL: unsupervised learning

## Data Availability

The survey and raw data used for this study are available as supplemental information.
